# Ist JAK-Hemmung eine Option in der Behandlung der interstitiellen Lungenerkrankung bei einer rheumatoiden Arthritis?

**DOI:** 10.1007/s00393-023-01434-2

**Published:** 2023-10-17

**Authors:** Tobias Hoffmann, Ulf Teichgräber, Bianca Lassen-Schmidt, Claus Kroegel, Martin Krämer, Martin Förster, Diane Renz, Peter Oelzner, Joachim Böttcher, Marcus Franz, Gunter Wolf, Felix Güttler, Alexander Pfeil

**Affiliations:** 1 Klinik für Innere Medizin III, Universitätsklinikum Jena – Friedrich-Schiller-Universität Jena, Am Klinikum 1, 07747 Jena, Deutschland; 2 Institut für Diagnostische und Interventionelle Radiologie, Universitätsklinikum Jena – Friedrich-Schiller-Universität Jena, Am Klinikum 1, 07747 Jena, Deutschland; 3 Fraunhofer Institut für Digitale Medizin MEVIS, Max-von-Laue-Str. 2, 28359 Bremen, Deutschland; 4 Klinik für Innere Medizin I, Universitätsklinikum Jena – Friedrich-Schiller-Universität Jena, Am Klinikum 1, 07747 Jena, Deutschland; 5 Institut für Diagnostische und Interventionelle Radiologie, Department für Kinderradiologie, Medizinische Hochschule Hannover, Carl-Neuberg-Str. 1, 30625 Hannover, Deutschland

**Keywords:** Rheumatoide Arthritis, Interstitielle Lungenerkrankung, JAK-Hemmung, Auf künstlicher Intelligenz basierende Quantifizierung der pulmonalen hochauflösenden Computertomographie, Quantitative Lungenbildgebung, Rheumatoid arthritis, Interstitial lung disease, JAK inhibition, Artificial intelligence-based quantification of pulmonary high-resolution computed tomography, Quantitative lung imaging

## Abstract

Ein 69-jähriger Patient mit einer seropositiven erosiven rheumatoiden Arthritis (RA) stellte sich aufgrund einer progredienten Dyspnoe in unserer Klinik vor. Im Rahmen der Diagnostik wurden mittels einer hochauflösenden Computertomographie (HRCT) und einer immunologischen bronchoalveolären Lavage Milchglastrübungen als auch eine lymphozytäre Alveolitis als Folge einer interstitiellen Lungenerkrankung (ILD) bei einer RA nachgewiesen. Unter Berücksichtigung der Vortherapien erfolgte die Umstellung der DMARD („disease-modifying antirheumatic drug“)-Therapie auf Tofacitinib. Die DMARD-Therapie mit Tofacitinib zeigte eine Reduktion der Milchglastrübungen mittels auf künstlicher Intelligenz basierender Quantifizierung der pulmonalen hochauflösenden Computertomographie um 33 % im Verlauf über 6 Monate, welche mit einer Verbesserung der Dyspnoesymptomatik assoziiert war. Zusammenfassend stellt Tofacitinib eine effektive antiinflammatorische Therapieoption in der Behandlung einer RA-ILD dar.

## Abstract (en)

A 69-year-old male patient with seropositive erosive rheumatoid arthritis (RA) presented to our clinic due to progressive dyspnea. High-resolution computed tomography (HRCT) and immunological bronchioalveolar lavage revealed ground-glass opacities and a lymphocytic alveolitis caused by interstitial lung disease (ILD) in RA. Considering previous forms of treatment, disease-modifying antirheumatic drug (DMARD) treatment was switched to tofacitinib. Tofacitinib treatment demonstrated a 33% reduction in ground-glass opacities by artificial intelligence-based quantification of pulmonary HRCT over the course of 6 months, which was associated with an improvement in dyspnea symptoms. In conclusion, tofacitinib represents an effective anti-inflammatory therapeutic option in the treatment of RA-ILD.

## Anamnese

Ein 69-jähriger Patient mit einer seropositiven erosiven rheumatoiden Arthritis (RA; Rheumafaktor und Antikörper gegen das citrullinierte Protein/Peptid positiv, Erstdiagnose Juni 2003) stellte sich aufgrund einer seit 5 Monaten progredienten Dyspnoe erstmals im Januar 2019 in unserer Klinik vor. Die Dyspnoe entsprach einer NYHA-Klasse II–III. Anamnestisch sind eine arterielle Hypertonie, eine chronisch obstruktive Lungenerkrankung (COPD) und eine Prostatahyperplasie bekannt. Bis 2008 konsumierte der Patient aktiv Nikotin (kumulativ 30 „pack years“). Bezüglich der RA erfolgte eine DMARD („disease-modifying antirheumatic drug“)-Therapie zwischen Juni 2003 und März 2004 mit Methotrexat, welche aufgrund von gastrointestinalen Nebenwirkungen beendet wurde. Von April 2004 bis Januar 2008 erfolgte eine Therapie mittels Adalimumab, welche bei rezidivierenden Infekten im Februar 2008 auf Etanercept umgestellt wurde.

## Klinische Untersuchung

In der klinischen Untersuchung zeigen sich keine Ödeme oder anderweitigen kardiopulmonalen Dekompensationszeichen. Klinisch präsentierten sich eine Arthritis und schmerzhafte Gelenke im Bereich der Radiokarpalgelenke beidseits, Metakarpophalangealgelenke II–V beidseits und proximalen Interphalangealgelenke II–IV beidseits.

## Diagnostik

### Labor

#### Klinische Chemie

C‑reaktives Protein (CRP): 61,1 mg/l (Referenz: < 5,0 mg/l), Blutsenkungsgeschwindigkeit (1./2. Stunde): 18/47 mm/h. Leberfermente und Nierenretentionswerte: normwertig.

#### Blutbild

Leukozyten 5,2 Gpt/l (Referenz: 4,4–11,3 Gpt/l), Thrombozyten 278 Gpt/l (Referenz: 150–360 Gpt/l), Erythrozyten 5,2 Gpt/l (Referenz: 4,5–5,93 Gpt/l), Hämoglobin 8,5 mmol/l (Referenz: 8,7–10,9 mmol/l).

### Aktivitätsparameter

Disease Activity Score 28 (CRP): 5,12.

### Echokardiographie

Gute linksventrikuläre Funktion ohne regionale Wandbewegungsstörungen. Erhaltene rechtsventrikuläre Funktion. Keine relevanten Klappenvitien. Normaler Füllungsdruck. Normaler systolischer pulmonalarterieller Druck. V. cava inferior schmal.

### Lungenfunktionsuntersuchung

In der Lungenfunktionsuntersuchung zeigte sich keine signifikante Einschränkung (< 80 %) der FEV_1_, FVC oder TLC. Jedoch wurde eine schwere Diffusionsstörung bei einer DLCO von 36,8 % nachgewiesen (Tab. 1).

**Tab. 1:** Verlauf der Lungenfunktionsparameter

Lungenfunktionsparameter	Baseline (in %)	Follow-up Monat 6 (in %)
Forcierte Vitalkapazität (FVC)	117,4	105,3
Einsekundenkapazität (FEV_1_)	97,2	78,6
Diffusionskapazität von Kohlenmonoxid (DLCO)	36,8	37,6
Totale Lungenkapazität (TLC)	111,9	102,0

### Hochauflösende Computertomographie (HRCT)

**Abb. 1:**
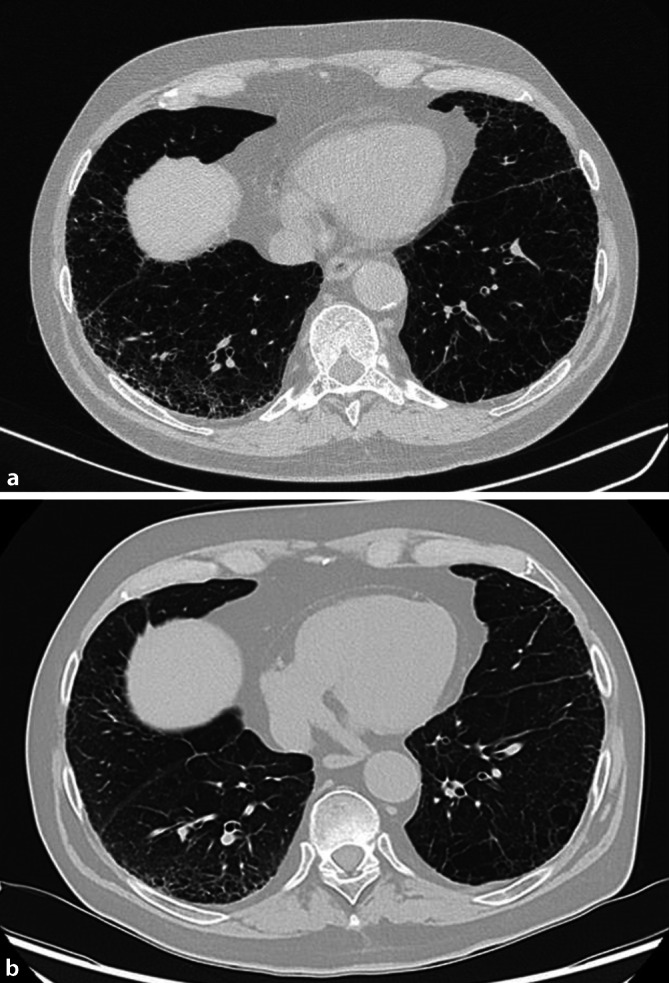
**a** Baseline: hochauflösende Computertomographie (HRCT) der Lunge mit Nachweis von Milchglastrübungen und Retikulationen. **b** Monat 6: hochauflösende Computertomographie (HRCT) der Lunge mit Nachweis einer Regredienz der Milchglastrübungen

Es stellen sich retikuläre Verdickungen der Septen mit Milchglastrübungen (Abb. 1a) sowie Honigwabenmuster („honey combing“) bildmorphologisch passend zu einer gewöhnlichen interstitiellen Pneumonie (UIP) dar.

### Auf künstlicher Intelligenz („artificial intelligence“) basierende Quantifizierung der pulmonalen hochauflösenden Computertomographie (AIqpHRCT)

**Abb. 2:**
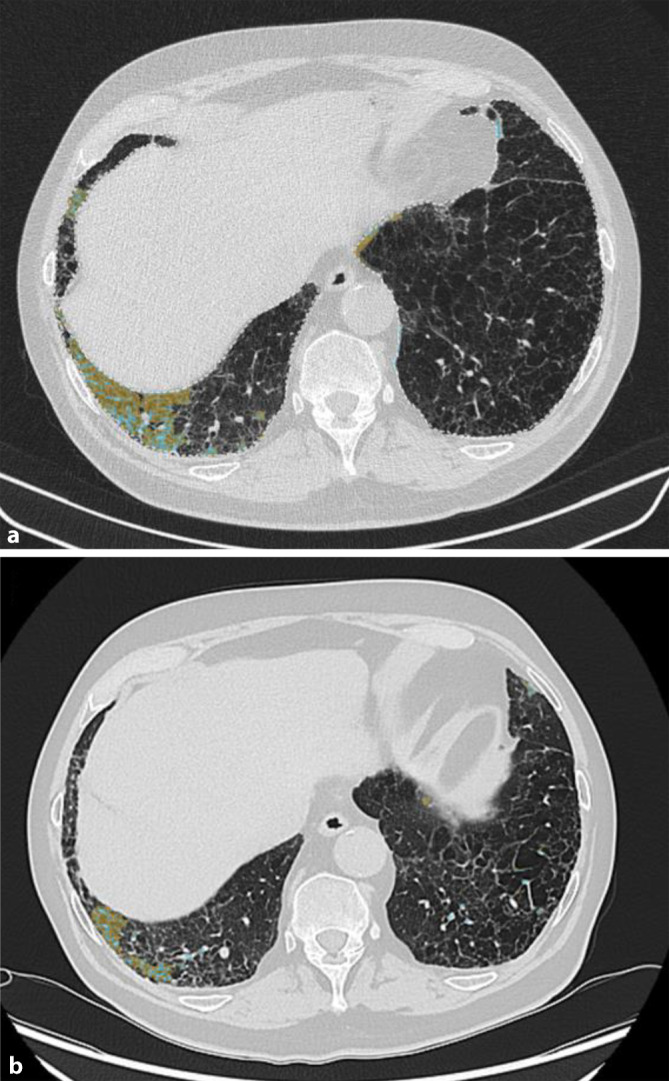
Künstliche Intelligenz basierende Quantifizierung der pulmonalen hochauflösenden Computertomographie („artificial intelligence-based quantification of pulmonary HRCT“ [AIqpHRCT]) der Milchglastrübungen **a** Baseline: 1,2 % und **b** Monat 6: 0,8 %

Die Quantifizierung der Milchglastrübungen und Retikulationen erfolgte durch die AIqpHRCT, einer KI (künstliche Intelligenz)-gestützten Annotationssoftware SATORI (RACOON Lung Analysis Platform Version 1.8.0, Fraunhofer, MEVIS, Bremen, Deutschland). AIqpHRCT konnte in 1,2 % des Lungenvolumens Milchglastrübungen aufzeigen (Abb. 2a). Der Anteil an fibrotischen Strukturen („high-attenuation areas“) lag bei 6,6 %.

### Bronchoskopie

#### Mikrobiologische bronchoalveoläre Lavage

Unauffällig.

#### Immunologische bronchoalveoläre Lavage

Deutliche Lymphozytose von 61 %, keine Neutrophilie. Es handelt sich um eine lymphozytäre Alveolitis. Der Befund könnte zu einer pulmonalen Beteiligung im Rahmen der rheumatoiden Arthritis passen.

## Diagnose

Interstitielle Lungenerkrankung (ILD) bei einer rheumatoiden Arthritis (RA).

## Therapie und Verlauf

Aufgrund der nachgewiesenen aktiven RA-ILD und unter Berücksichtigung der rheumatologischen Medikamentenanamnese erfolgte die Umstellung der Basistherapie von Etanercept auf Tofacitinib. Hierunter wies der Patient eine Regredienz der Krankheitsaktivität (Disease Activity Score 28 [CRP]: 1,9) und der Dyspnoesymptomatik (NYHA I–II) auf. Eine Änderung der Lungenfunktionsparameter trat nicht auf (Tab. 1). In der Verlaufskontrolle nach 6 Monaten konnte im HRCT eine Abnahme der Milchglastrübungen dargestellt werden (Abb. 2b). In der KI-basierten Quantifizierung der Milchglastrübungen im HRCT mittels AIqpHRCT wurde eine Reduktion des Volumens der Milchglastrübungen um 33 % nachgewiesen (Abb. 2b und 3). Das Honigwabenmuster wies keine Änderung im Verlauf auf. Der Anteil an fibrotischen Strukturen („high-attenuation areas“) lag annähernd konstant bei 6,8 %.

**Abb. 3:**
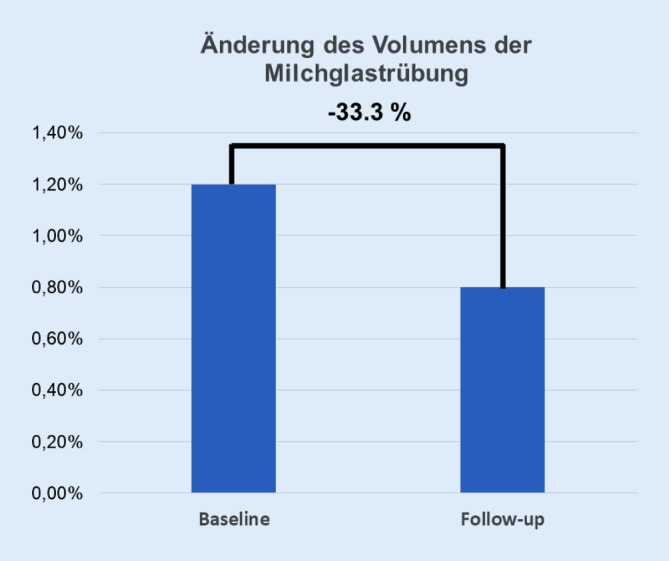
Darstellung der Abnahme der Milchglastrübungen mittels auf künstlicher Intelligenz basierender Quantifizierung der pulmonalen hochauflösenden Computertomographie („artificial intelligence-based quantification of pulmonary HRCT“ [AIqpHRCT]) im Verlauf über 6 Monate unter der Therapie mit Tofacitinib

## Diskussion

Eine RA-ILD wird bei ca. 10 % der Patienten diagnostiziert [1], wobei bei 34 % eine ILD 1 Jahr vor der Diagnosestellung einer RA oder im Jahr der RA-Erstdiagnose nachgewiesen wird [2]. Dabei ist die RA-ILD mit einer erhöhten Mortalität (5-Jahres-Mortalität RA-ILD 39,0 % vs. RA ohne ILD 18,2 %) verbunden [2], wobei die verzögerte Diagnosestellung der RA-ILD auch zu einer erhöhten Mortalität führt [3].

Ein Screeningalgorithmus zur Detektion einer RA-ILD ist klinisch nicht etabliert. Möglicherweise stellt der Abfall der DLCO < 80 % einen sensitiven Parameter zur Detektion einer ILD dar [4]. Bildgebend ist das HRCT der Goldstandard zur Darstellung einer RA-ILD [5]. Dabei können Milchglastrübungen im HRCT das bildmorphologische Korrelat einer immunologischen lymphozytären Alveolitis darstellen [6].

Rituximab und Abatacept zeigten einen positiven Einfluss auf die Behandlung einer RA-ILD [7, 8]. Limitierend für die Therapie mit Rituximab und Abatacept ist, dass nur eine Zulassung für die Kombinationstherapie mit Methotrexat in der Behandlung der RA vorliegt. Aufgrund der Methotrexat-Unverträglichkeit bei dem dargestellten Fall stellt die Anwendung von Rituximab und Abatacept in der Monotherapie einen Off-label-Use dar. Aus diesem Grund bestand die Indikation zur Einstellung auf ein „disease-modifying antirheumatic drug“, welches in der Monotherapie zugelassen ist. Eine solche Therapiealternative stellt der Inhibitor der Januskinase Tofacitinib dar.

Eine kürzlich publizierte retrospektive Studie zeigte, dass für Tofacitinib die geringste Inzidenzrate (1,47) im Vergleich zu Adalimumab (3,43), Abatacept (4,46), Rituximab (6,15) und Tocilizumab (5,05) für das Auftreten einer RA-ILD vorliegt [9]. In einer weiteren initialen Studie von Kalyoncu et al. konnte für Tofacitinib im Median über 12 Monate keine signifikante Änderung der Lungenfunktionsparameter (FVC und FEV_1_ %) bei einer RA-ILD nachgewiesen werden [10]. Anhand des dargestellten Patientenfalles kann ein gleichsinniges Ergebnis für die Lungenfunktion evaluiert werden. Zusätzlich erfolgte die Durchführung einer HRCT-Verlaufskontrolle, bei der eine Abnahme des Volumens der Milchglastrübungen um 33 % mittels AIqpHRCT, einer neuen KI-basierten Analysetechnik für das HRCT, erstmalig nachweisbar war. Klinisch zeigte der Patient eine Verbesserung der Dyspnoesymptomatik, sodass eine direkte Korrelation der Klinik zur AIqpHRCT des Thorax bestanden hat. Die deutliche Abnahme der Milchglastrübungen in der HRCT, welche das bildmorphologische Korrelat einer immunologischen lymphozytären Alveolitis widerspiegeln kann [6], ist möglicherweise auf eine breite immunsuppressive Wirkung in Form einer Multizytokinhemmung von Januskinasehemmern zurückzuführen [11].

Zur Behandlung der UIP-Komponente, welche bildmorphologisch durch ein Honigwabenmuster und histologisch überwiegend durch eine Fibrose gekennzeichnet ist [5], ist die Erweiterung der Therapie um den antifibrotischen Wirkstoff wie Nintedanib indiziert [12, 13], welcher zum Behandlungszeitpunkt des Patienten noch nicht für die Therapie der RA-ILD zugelassen war.

Die AIqpHRCT stellt aktuell eine Forschungssoftware dar, welche zur Detektion und Quantifizierung von Lungenparenchymveränderungen bei einer ILD genutzt werden kann. Perspektivisch wird der klinische Einsatz der AIqpHRCT als KI-gestützte Annotationssoftware zur Auswertung der HRCT geplant, sodass mittels AIqpHRCT eine Verlaufsbeurteilung der ILD bei einer RA bzw. entzündlich rheumatischen Erkrankungen im klinischen Alltag möglich ist. Bezüglich der Kosten und Vergütung der AIqpHRCT in der klinischen Routine können zum jetzigen Zeitpunkt leider keine Aussagen getroffen werden.

Anhand dieses Falles kann erstmalig gezeigt werden, dass der Einsatz eines Januskinaseinhibitors zu einer Reduktion der Milchglastrübungen und somit der RA-ILD im HRCT führt und dieser Sachverhalt mittels der AIqpHRCT analysierbar ist. In weiteren prospektiven Studien ist die Effektivität einer Januskinasehemmung zur Behandlung einer RA-ILD zu verifizieren und die klinische Wertigkeit der KI-basierten Quantifizierung der Lungenparenchymveränderungen mittels AIqpHRCT im HRCT bei einer RA-ILD zu evaluieren.

## Fazit für die Praxis

- Eine RA-ILD mit Milchglastrübungen im HRCT kann in der immunologischen bronchoalveolären Lavage durch eine lymphozytäre Alveolitis gekennzeichnet sein.
- Die Quantifizierung der Milchglastrübungen ist durch eine KI-gestützte Analyse des HRCT mittels AIqpHRCT möglich.
- Tofacitinib stellt eine effektive antiinflammatorische Therapie der RA-ILD dar.

## Abkürzungen

AIqpHRCT: Artificial intelligence (künstliche Intelligenz) basierende Quantifizierung der pulmonalen hochauflösenden Computertomographie
CRP: C‑reaktives Protein
DLCO: Diffusionskapazität von Kohlenmonoxid
DMARD: Disease-modifying antirheumatic drugs
HRCT: Hochauflösende Computertomographie
ILD: Interstitielle Lungenerkrankung
KI: Künstliche Intelligenz
NSIP: Nichtspezifische interstitielle Pneumonie
RA: Rheumatoide Arthritis
UIP: Gewöhnliche interstitielle Pneumonie („usual interstitial pneumonia“)
